# Late Inflammation Induced by Asbestiform Fibers in Mice Is Ameliorated by a Small Molecule Synthetic Lignan

**DOI:** 10.3390/ijms222010982

**Published:** 2021-10-12

**Authors:** Reagan Badger, Kyewon Park, Ralph A. Pietrofesa, Melpo Christofidou-Solomidou, Kinta M. Serve

**Affiliations:** 1 Department of Biological Sciences, Idaho State University, Pocatello, ID 83209, USA; reaganbadger@isu.edu; 2 Division of Pulmonary, Allergy, and Critical Care, Perelman School of Medicine, University of Pennsylvania, Philadelphia, PA 19104, USA; kyewpark@pennmedicine.upenn.edu (K.P.); ralphp@pennmedicine.upenn.edu (R.A.P.); melpo@pennmedicine.upenn.edu (M.C.-S.)

**Keywords:** asbestiform fibers, asbestos, autoimmunity, inflammation, LGM2605, Libby amphibole, secoisolariciresinol diglucoside

## Abstract

Exposure to Libby amphibole (LA) asbestos-like fibers is associated with increased risk of asbestosis, mesothelioma, pulmonary disease, and systemic autoimmune disease. LGM2605 is a small molecule antioxidant and free radical scavenger, with anti-inflammatory effects in various disease models. The current study aimed to determine whether the protective effects of LGM2605 persist during the late inflammatory phase post-LA exposure. Male and female C57BL/6 mice were administered daily LGM2605 (100 mg/kg) via gel cups for 3 days before and 14 days after a 200 µg LA given via intraperitoneal (i.p.) injection. Control mice were given unsupplemented gel cups and an equivalent dose of i.p. saline. On day 14 post-LA treatment, peritoneal lavage was assessed for immune cell influx, cytokine concentrations, oxidative stress biomarkers, and immunoglobulins. During the late inflammatory phase post-LA exposure, we noted an alteration in trafficking of both innate and adaptive immune cells, increased pro-inflammatory cytokine concentrations, induction of immunoglobulin isotype switching, and increased oxidized guanine species. LGM2605 countered these changes similarly among male and female mice, ameliorating late inflammation and altering immune responses in late post-LA exposure. These data support possible efficacy of LGM2605 in the prolonged treatment of LA-associated disease and other inflammatory conditions.

## 1. Introduction

From the 1920s through 1990, approximately 80% of the world’s vermiculite was mined and shipped from Libby (MT, USA). Vermiculite is a naturally occurring mineral that expands when heated and is used for various applications, including home insulation. Unfortunately, the vermiculite mined from Libby was contaminated with amphibole asbestos and asbestiform fibers, including a mixture of winchite, richterite, and tremolite fibers [1,2]. While Libby amphibole (LA) exposures were most significant among vermiculite mine and processing plant workers, they occurred anywhere the vermiculite was distributed, most notably as Zonolite Attic Insulation [3].

Exposures to LA and similar asbestiform amphibole fibers have been linked to development of asbestos related diseases (ARDs), including asbestosis and mesothelioma [4,5,6,7,8,9,10], and lamellar pleural thickening, leading to progressive loss of lung function and increased mortality rates [11,12]. While lung diseases are the predominant outcome of asbestos exposure, ARDs include various systemic disorders like gastrointestinal tract cancers, ovarian cancer, and pericardial effusions. Additionally, LA has been linked to development of autoimmune responses, characterized by increased prevalence of anti-nuclear antibodies (ANAs) and cell-specific autoantibodies as well as increased incidence of systemic autoimmune diseases [13,14,15]. While the etiology of amphibole-associated disease is undoubtedly complex and multifaceted, unresolved inflammation and oxidative damage have been linked to pathogenesis [11,14,16].

Following inhalation, LA fiber deposition initiates a complex set of inflammatory cascades beginning with activation of resident lung cells (alveolar, mesothelial, fibroblast) followed by recruitment of circulating macrophages and polymorphonuclear (PMN) leukocytes [17,18,19,20]. These cells influx to the site of fiber deposition where they release reactive oxygen species (ROS) [21,22,23], activate NLRP3 inflammasomes [24,25], and produce the pro-inflammatory cytokines IL-1β, IL-18, and TNF-α [26], thus driving a feed-forward loop of inflammation and immune cell activation [27,28].

Additionally, following deposition in lungs, asbestos fibers can translocate to the pleural cavity, causing injury to mesothelial cells and resulting in immune cell recruitment. We, and others, have linked asbestos-associated immune responses, particularly autoimmunity, to a progressive form of pleural fibrosis [11,16,29]. Since non-resolution of inflammation is thought to contribute to development of autoimmunity and ARDS, we predicted that blocking early inflammation and reducing oxidative stress responses would provide therapeutic benefits by limiting immune cell recruitment to sites of LA fiber deposition.

Recent reports describe the usefulness of LGM2605, a synthetic secoisolariciresinol diglucoside (SDG) or flaxseed lignan, in preventing amphibole asbestos-induced cytotoxicity, inflammation, and oxidative stress, both in vitro and in vivo [22,30,31]. Mechanisms by which LGM2605 protects against asbestos-induced cytotoxicity include reduction of NLRP3 inflammasome activity [22] and upregulation of the Nrf2/ARE antioxidant pathway [32]. Additionally, LGM2605 has been shown to protect against oxidative stress in an Nrf2-independent manner by acting as a direct free radical scavenger [33] and a direct scavenger of active chlorine species such as those generated from activated myeloperoxidase, an enzyme prevalent in immune cells such as neutrophils and macrophages [34,35,36].

Our most recent work examined the ability of LGM2605 to mitigate hyper-acute immune responses to LA exposure in a C57BL/6 mouse model [37]. LGM2605 treatment (100 mg/kg daily via oral gavage) significantly reduced immune cell recruitment to the peritoneal cavity within 3 days of a single bolus intraperitoneal dose of LA (200 µg). The peritoneal cavity was used as a surrogate for the pleural cavity due to its similar cellular composition, as previously described [38,39]. Specifically, LGM2605 significantly attenuated LA-induced splenomegaly and recruitment of macrophage and PMN cells [37]. These data suggest that LGM2605 protects against very early innate immune events induced by LA exposure. However, we did not examine the effects of LGM2605 on resident peritoneal cells (i.e., mesothelial cells) nor were we able to elucidate effects of adaptive immune responses due to the short experimental duration (3 days). This current study expands upon our previous work by demonstrating an in vivo LGM2605-mediated reduction in inflammatory cytokine production and innate immune cell recruitment at the site of fiber deposition. We further demonstrate that LA exposure altered lymphocyte levels and immunoglobulin isotype distributions and that LGM2605 treatment restored these to control levels. Lastly, we show that LGM2605 reduces LA-associated oxidative stress production within the peritoneal cavity. Overall, our findings show that oral consumption of the flaxseed derivative SDG limits late acute-phase responses to asbestos exposure, suggesting a convenient and effective strategy to mitigate systemic and local responses to fiber exposure.

## 2. Results

### 2.1. Mice Consumed Approximately 100 mg/kg LGM2605 Daily 

**Figure 1:**
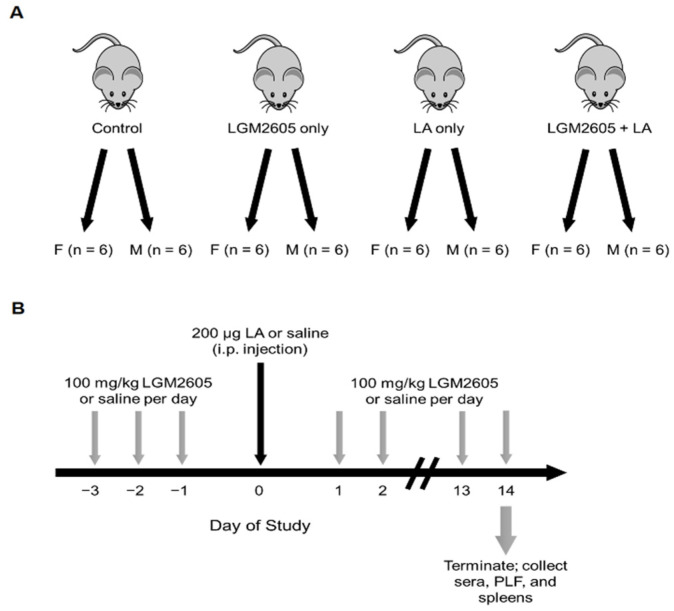
Assignment of treatment groups and schedule of treatment. (**A**) Mice were randomly divided into one of 4 treatment groups, with 6 males and 6 females per group. Each treatment is designated by diet/i.p. injection: control (saline/saline), LGM2605 only (LGM2605/saline), LA only (saline/LA), and LGM2605 + LA (LGM2605/LA). (**B**) For 3 days prior to i.p. injection, mice received daily treatment of LGM2605 or saline control via Medigel cups. LA fibers or saline control were then delivered by i.p. injection. Mice received daily treatment of LGM2605 or saline control for an additional 14 days following LA exposure. On day 14, mice were euthanized, and tissues were collected for subsequent analysis.

To examine the effect of LGM2605 on adaptive immune responses, we used a mouse model of LA exposure and LGM2605 treatment similar to that previously described [37]. However, previous experiments relied on oral gavage for LGM2605 administration; this approach is time consuming for researchers and stressful for animals over longer experimental durations. Therefore, in the current study, we opted to use Medigel cups for drug delivery, as described in detail in Section 4.2. Briefly, mice (*n* = 48) were divided into four treatment groups, consisting of equal numbers male and female animals (Figure 1A). Mice were treated daily with approximately 100 mg/kg LGM2605 or saline (control). Drug or control were administered for 3 days prior and 14 days following a single bolus intraperitoneal (i.p.) injection of LA fibers (200 µg) or saline (Figure 1B).

Medigel cups were weighed at the start and end of each 24-h period to measure mouse consumption. Average consumption of drug was calculated based on individual mouse body weights and was not altered by presence of food dye (used to ensure even distribution of drug throughout the gel). The average daily amount of drug consumed was 104.3 mg/kg for females (CI95% ± 2.32) and 101.9 mg/kg for males (CI95% ± 3.23). There was no statistically significant difference between male and female consumption. Control mice were fed unsupplemented Medigel cups containing an equivalent dose of saline (vehicle). Overall, mouse weights remained constant over the course of the study, with an average daily weight change of +0.06 g (CI95% ± 0.02), suggesting adequate hydration and weight gain. 

### 2.2. LGM2605 Treatment Mitigated Innate Immune Responses to LA

**Figure 2:**
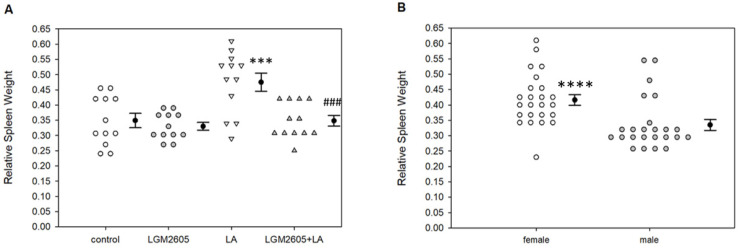
Spleen weight analysis. Relative spleen weights were determined by dividing raw spleen weights by individual mouse body weights. (**A**) LA treatment significantly increased relative spleen weights compared to saline control, *** *p* = 0.001; LGM2605 + LA treatment reduced relative spleen weights back to control levels, ### *p* < 0.001. Following two-way ANOVA, data were analyzed via Tukey post-hoc test. The observed patterns were consistent with sex; however, female mice demonstrated significantly higher relative spleen weights compared to males (**B**), **** *p* < 0.0001 by one-way ANOVA.

To verify that LGM2605 suppressed innate immune responses up to 14 days post-LA exposure, we analyzed relative spleen weights and leukocyte influx into the peritoneal cavity. Relative spleen weights were calculated by dividing the spleen weight by total mouse weight. Overall, relative spleen weights were significantly altered by treatment, *p* < 0.0001 by two-way ANOVA. LA treatment significantly increased relative spleen weights compared to control, *p* = 0.001 by Tukey post-hoc test (Figure 2A). Treatment with LGM2605 + LA reduced relative spleen weight back to control levels, *p* < 0.001 by Tukey post-hoc test. Female mice demonstrated significantly higher relative spleen weights compared to males, *p* < 0.0001 by two-way ANOVA (Figure 2B), but the overall pattern of LA-induced increases and LGM2605-induced decreases in splenomegaly were consistent within each sex.

**Figure 3:**
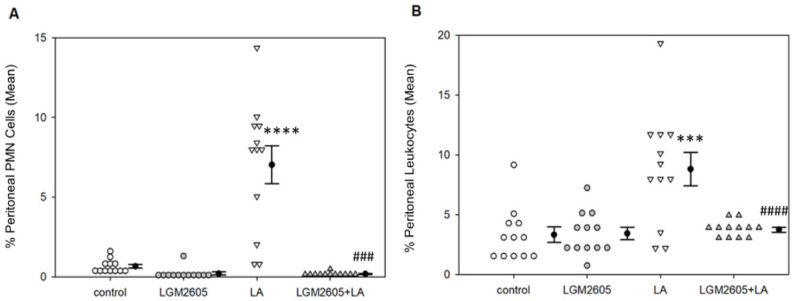
Detection of peritoneal PMN cells and leukocytes by flow cytometry. (**A**) LA treatment significantly increased the percentage of peritoneal PMNs compared to saline control, **** *p* < 0.0001; LGM2605 + LA treatment reduced the percentage of peritoneal PMNs back to control levels, ### *p* < 0.001. (**B**) LA treatment significantly increased the percentage of peritoneal compared to saline control, *** *p* < 0.001; LGM2605 + LA treatment reduced the percentage of peritoneal back to control levels, #### *p* < 0.0001. Following two-way ANOVA, data were analyzed via Tukey post-hoc test.

LGM2605 treatment significantly mediated LA-induced white blood cell (WBC) influx into the peritoneal cavity, similar to changes previously noted at 3 days post-LA exposure. Overall effects were significant among peritoneal PMN cells, *p* < 0.0001 by two-way ANOVA. Specifically, LA treatment significantly increased the percentage of peritoneal PMN cells (as a percent of total cells) compared to saline control, *p* < 0.0001 by Tukey post-hoc test (Figure 3A). Treatment with LGM2605 + LA reduced the percentage of peritoneal PMN cells back to control levels, *p* < 0.001 by Tukey post-hoc test. Treatment effects were also significant among peritoneal macrophages, *p* < 0.0001 by two-way ANOVA. LA treatment significantly increased the percentage of peritoneal leukocytes compared to saline control, *p* < 0.001 by Tukey post-hoc test (Figure 3B). Treatment with LGM2605 + LA reduced the percentage of peritoneal back to control levels, *p* < 0.0001 by Tukey post-hoc test. No sex effect was noted for WBC cells in PLF, and no differences in splenic WBC numbers were detected among any treatment groups (data not shown).

### 2.3. LGM2605 Treatment Alters Peritoneal and Splenic B1a B Cell Levels Following LA Exposure

**Figure 4:**
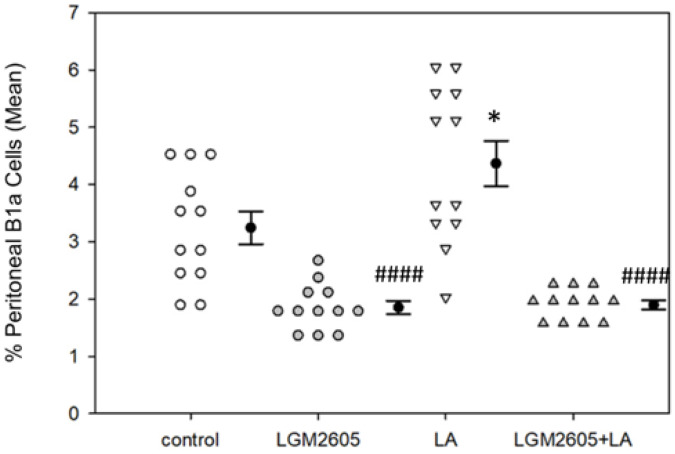
Detection of peritoneal B1a B cells by flow cytometry. LA treatment significantly increased the percentage of peritoneal B1a B cells compared to saline control, * *p* = 0.017. LGM2605 treatment, either alone or combined with LA, significantly reduced the percentage of peritoneal B1a B cells compared to both LA only (#### *p* < 0.0001) and saline control (#### *p* < 0.01). Following two-way ANOVA, data were analyzed via Tukey post-hoc test. No sex effects were observed.

As a measure of local immune cell activation, peritoneal and splenic B1a B cell numbers were assessed. Overall, treatment effects were significant among peritoneal B1a B cells, *p* < 0.0001 by two-way ANOVA. Treatment with LA alone significantly increased peritoneal B1a B cells (as a percent of total lymphocytes) compared to saline control, *p* = 0.017 by Tukey post-hoc test (Figure 4). 

**Figure 5:**
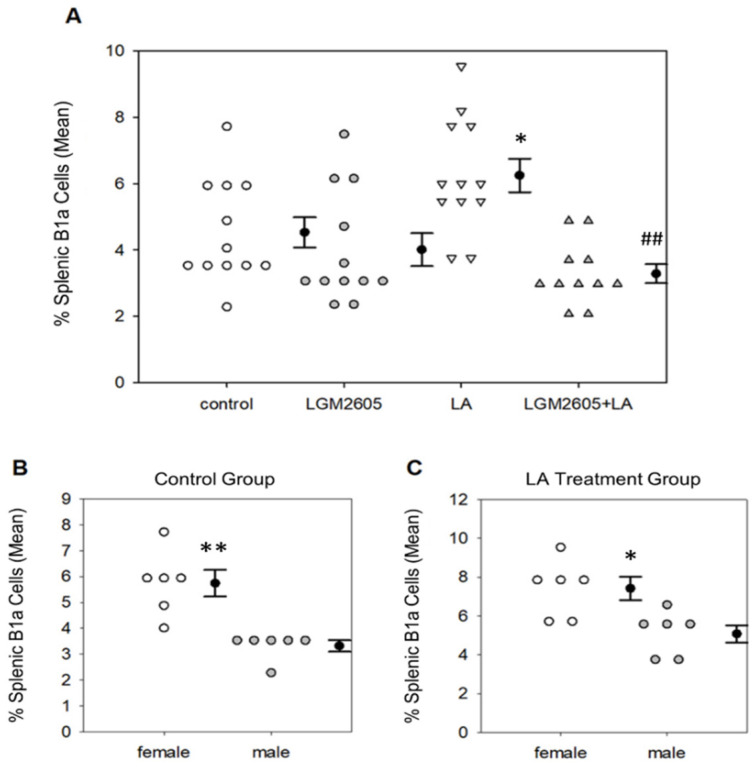
Detection of splenic B1a B cells by flow cytometry. (**A**) LA treatment significantly increased the percentage of splenic B1a B cells compared to saline control, * *p* = 0.043; LGM2605 + LA treatment reduced the percentage of splenic B1a B cells back to control levels, ## *p* < 0.01 as compared to LA treatment. Following two-way ANOVA, data were analyzed via Tukey post-hoc test. Female mice had a significantly higher percentage of splenic B1a B cells compared to males in both the control group (**B**), ** *p* < 0.01 by one-way ANOVA, and the LA only group (**C**), * *p* = 0.015 by one-way ANOVA.

Interestingly, LGM2605 treatment, either alone or combined with LA, significantly reduced the percentage of peritoneal B1a B cells compared to both LA only (*p* < 0.0001 by Tukey post-hoc test) and control (*p* < 0.01 by Tukey post-hoc test). Treatment effects were also significant among splenic B1a cells, *p* < 0.0001 by two-way ANOVA. LA treatment significantly increased the percentage of splenic B1a cells compared to saline control, *p* = 0.043 by Tukey post-hoc test (Figure 5A). LGM2605 treatment, either alone or combined with LA, significantly reduced the percentage of B1a cells back to control levels, *p* < 0.01 by Tukey post-hoc test. No effect of sex was determined for peritoneal B1a B cell populations, though there was a significant sex effect for splenic B1a B cells, *p* < 0.01 by two-way ANOVA (Figure 5B). Female mice had a significantly higher percentage of splenic B1a B cells compared to males in the control group (*p* < 0.01 by one-way ANOVA) and in the LA only group (*p* = 0.015 by one-way ANOVA). However, this difference was not detected in the LGM2605 treatment groups.

### 2.4. LGM2605 Treatment Alters Adaptive Immune Responses Following LA Exposure

**Figure 6:**
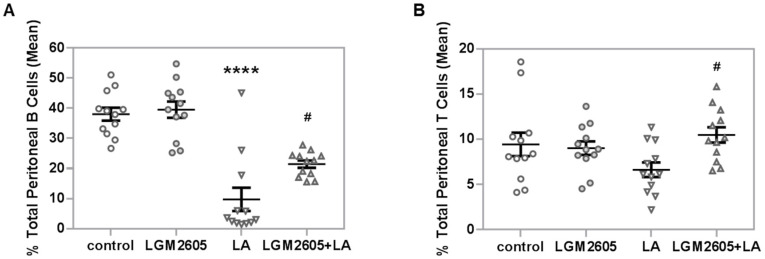
Detection of total peritoneal B and T cells by flow cytometry. (**A**) LA treatment significantly reduced the percentage of total peritoneal B cells compared to saline control, **** *p* < 0.0001. LGM2605 + LA treatment partially restored the percentage of total peritoneal B cells (*p* = 0.017), though they were still significantly lower than saline control or LGM2605 only (*p* < 0.001). (**B**) LGM2605 + LA treatment restored the percentage of total peritoneal T cells to control levels, # *p =* 0.0317. No effect of sex was noted for B or T cells. Following two-way ANOVA, data were analyzed via Tukey post-hoc test.

To examine the effect of LGM2605 on LA-induced adaptive immune responses, peritoneal and splenic lymphocytes were measured. Overall, treatment effects were significant among total peritoneal B and T cells, with LA treatment reducing total peritoneal B and T cells compared to saline control (Figure 6A,B). Treatment with LGM2605 + LA partially restored the percentage of total peritoneal B cells (*p* = 0.17 by Tukey post-hoc test), though they were still significantly lower than saline control or LGM2605 alone *(p* < 0.001 by Tukey post-hoc test). LGM2605 treatment returned T cell levels back to control levels (*p* = 0.0317). No sex effect was detected among the B or T cells. Since this analysis was done using total cell numbers, it is possible that decreased lymphocyte numbers were due to the overall increase in total peritoneal cells resulting from the influx of white blood cells into the cavity. Therefore, we also analyzed B and T cell numbers as a percentage of peritoneal lymphocytes instead of as a percentage of total cells. The trends were the same as noted for total B and T cell populations, with no effect of sex (data not shown). Additionally, we found no effect of LA treatment on peritoneal Th cells (data not shown). Though not significant, a trend was noted in which LA treatment reduced Th numbers and treatment with LGM2605 returned these populations to control levels. In addition, no effects of LA were noted on the total splenic B or T cell populations nor on splenic Th cell populations (data not shown).

### 2.5. LGM2605 Treatment Significantly Alters Immunoglobulin Isotypes in PLF Following LA Exposure

**Figure 7:**
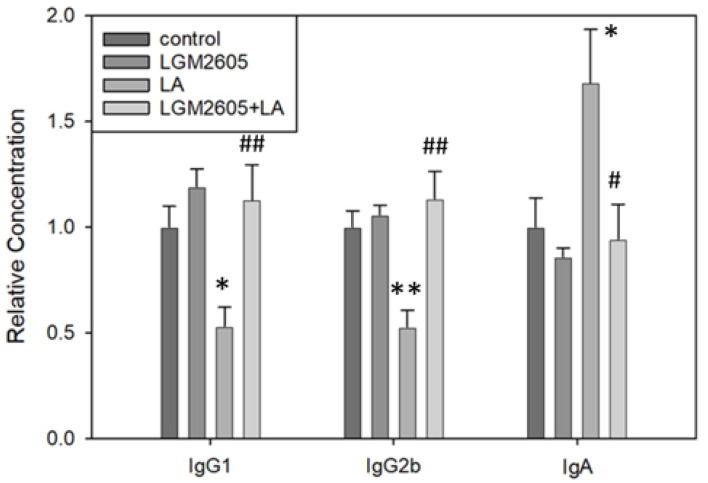
Detection of immunoglobulin isotypes in PLF via cytometry bead array. LA treatment significantly decreased the relative concentrations of IgG1 (* *p* = 0.032) and IgG2b (** *p* < 0.01) compared to saline control; LGM2605 + LA treatment significantly increased the concentrations of IgG1 (## *p* < 0.01) and IgG2b (## *p* < 0.01) back to control levels. LA exposure also significantly increased IgA concentration compared to saline control, * *p* = 0.037; LGM2605 + LA treatment reduced IgA concentration back to control levels, # *p* = 0.025. Following two-way ANOVA, data were analyzed via Tukey post-hoc test.

To further examine the adaptive immune responses, immunoglobulin isotypes were measured in PLF. Table 1 reports overall treatment effects and data show that LA treatment significantly decreased the relative concentrations of IgG_1_ (*p* = 0.032 by Tukey post-hoc test) and IgG_2b_ (*p* < 0.01 by Tukey post-hoc test) in PLF compared to saline control (Figure 7). Treatment with LGM2605 + LA significantly increased the concentrations of IgG_1_ (*p* < 0.01 by Tukey post-hoc test) and IgG_2b_ (*p* < 0.01 by Tukey post-hoc test) in PLF compared to LA treatment, thus returning concentrations back to control levels. In addition, LA treatment significantly increased the relative concentration of IgA in PLF compared to saline control, *p* = 0.037 by Tukey post-hoc test; treatment with LGM2605 + LA significantly decreased the relative concentration of IgA back to control levels, *p* = 0.025 by Tukey post-hoc test. Interestingly, there was a significant sex effect for IgA (*p* = 0.020 by two-way ANOVA), with control males demonstrating significantly higher IgA levels compared to control females (*p* < 0.01 by one-way ANOVA). No other sex effects were noted. In general, LA treatment decreased the ratio of κ antibody concentration/λ antibody concentration compared to saline control (Table 1). Treatment with LGM2605 + LA restored κ/λ ratios back to control levels. Neither LA nor LGM2605 + LA significantly altered the relative concentrations of IgG_2a_, IgG_3_, IgM, or IgE; however, trends in κ/λ ratios were consistent across all isotypes. Together, the lymphocyte and isotype data suggest B cell migration and activity were significantly affected by LA and that this effect was ameliorated by LGM2605.

### 2.6. LGM2605 Treatment Significantly Alters IL-6 and MCP-1 Concentrations in PLF and IL-6 and IL-10 Concentrations in Serum Following LA Exposure

**Figure 8:**
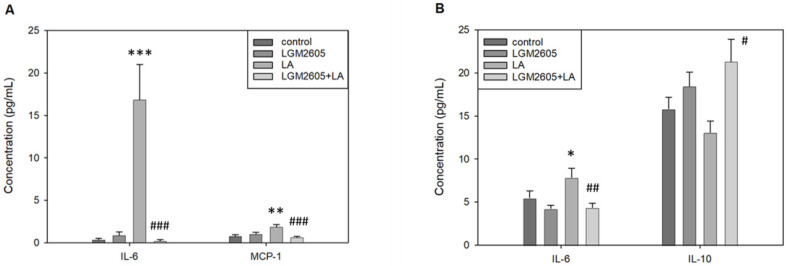
Detection of cytokines in PLF and sera by cytometric bead array. (**A**) In PLF, LA treatment significantly increased the concentrations of IL-6 (*** *p* < 0.001) and MCP-1 (** *p* < 0.01) compared to saline control; LGM2605 + LA treatment reduced the concentrations of IL-6 (### *p* < 0.001) and MCP-1 (### *p* < 0.001) back to control levels. (**B**) In sera, LA treatment significantly increased IL-6 concentration compared to saline control, * *p* = 0.015; LGM2605 + LA treatment reduced IL-6 concentration back to control levels, ## *p* < 0.01. LGM2605 + LA treatment also significantly increased IL-10 concentration in sera compared to LA only, # *p* = 0.011; however, neither LA nor LGM2605 + LA led to significant alterations in IL-10 concentration compared to saline control. Following two-way ANOVA, data were analyzed via Tukey post-hoc test.

To further examine immune activity, cytokine levels were assessed in both PLF and serum. Table 2 reports PLF cytokine concentrations that were significantly altered by treatment. LA treatment significantly increased the concentration of IL-6 in PLF compared to saline control, *p* < 0.001 by Tukey post-hoc test; the increase in IL-6 following LA exposure was more than 44-fold compared to saline control, which was the largest increase among any of the cytokines assessed. Treatment with LGM2605 + LA significantly reduced the concentration of IL-6 in PLF back to control levels, *p* < 0.001 by Tukey post-hoc test (Figure 8A). In addition, LA treatment significantly increased the concentration of MCP-1 in PLF compared to saline control, *p* < 0.01 by Tukey post-hoc test; treatment with LGM2605 + LA significantly reduced the concentration of MCP-1 in PLF back to control levels, *p* < 0.001 by Tukey post-hoc test. PLF concentrations of IL-10, TNF, IFN-γ, and IL-12p70 were also assessed but were unaffected by either LA or LGM2605 treatment at this time point. No effect of sex was detected among any of the cytokines examined.

Table 3 reports serum cytokine concentrations significantly altered by treatment. LA treatment significantly increased IL-6 concentration in serum compared to saline control, *p* = 0.015 by Tukey post-hoc test (Figure 8B). Treatment with LGM2605 + LA reduced IL-6 concentration back to control levels, *p* < 0.01 by Tukey post-hoc test. In addition, treatment with LGM2605 + LA significantly increased IL-10 concentration in serum compared to LA only, *p* = 0.011 by Tukey post-hoc test. However, neither LA nor LGM2605 + LA led to significant alterations in IL-10 concentration relative to control. Serum concentrations of MCP-1, TNF, IFN-γ, and IL-12p70 were also assessed but were unaffected by either LA or LGM2605 treatment at this time point. Notably, two-way ANOVA was significant for MCP-1 (as shown in Table 2), but Tukey post-hoc test revealed treatment effects to be insignificant. No effect of sex was detected among any of the cytokines examined.

### 2.7. LGM2605 Treatment Reduces Oxidative Stress Biomarkers in PLF Following LA Exposure

**Figure 9:**
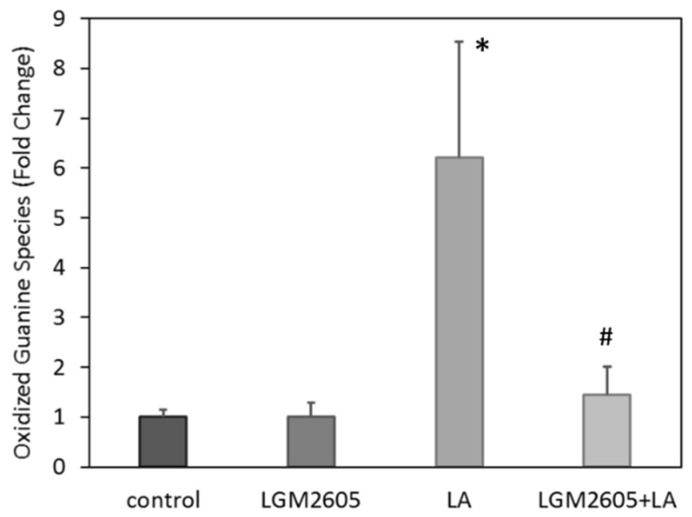
Detection of oxidized guanine species in PLF. LA treatment significantly increased relative concentrations of oxidized guanine species relative to saline control, * *p* = 0.023. LGM2605 + LA treatment decreased relative concentrations of oxidized guanine species back to control levels, # *p* = 0.022. Following two-way ANOVA, data were analyzed via Tukey post-hoc test. Data are reported as the mean fold change ± SEM in the levels of oxidized guanine species from their respective, non-LA-exposed controls, in PLF.

Overall, treatment effects were significant among levels of oxidized guanine species in PLF, *p* = 0.027 by one-way ANOVA (Figure 9). LA treatment significantly increased the relative concentrations of oxidized guanine species in PLF compared to saline control, (*p* = 0.023 by Tukey post-hoc test). Treatment with LGM2605 + LA significantly reduced the relative concentrations of oxidized guanine species compared to LA treatment (*p* = 0.022 by Tukey post-hoc test), returning concentrations back to control levels. No statistically significant effect of sex was measured.

## 3. Discussion

Libby amphibole (LA) asbestos is highly inflammatory and has been linked to the development of various asbestos-related diseases (ARDs), as well as autoimmune disease [14,29,40]. Development of ARDs and autoimmunity may be due to chronic, non-resolving inflammation and immune activation perpetuated by LA fiber deposition. Inhalation of LA fibers promotes increased trafficking of innate immune cells (i.e., PMN cells and macrophages) to the site of fiber deposition, where these cells release reactive oxygen species (ROS) [21,22,23], activate NLRP3 inflammasomes [24,25], and produce pro-inflammatory cytokines [25], contributing to oxidative stress and inflammation which drive further immune cell activation [26,27]. A potent antioxidant and free radical scavenger, synthetic LGM2605 has been proposed as a possible chemopreventive agent for targeting many of these early immune responses in order to reduce progression to chronic, less treatable disease states [41]. Prophylactic administration of natural compounds and derivatives have similarly been shown to protect against fibrosis, atherosclerosis, and oxidative damage [42,43,44].

LGM2605 has been shown to reduce asbestos-induced cytotoxicity via reduction of NLRP3 inflammasome activity [22] and upregulation of the Nrf2/ARE antioxidant pathway; it also reduces oxidative stress through its direct free radical-scavenging abilities. Our most recent work demonstrated LGM2605-induced reduction in inflammation and innate immune cell activation in a hyper-acute murine model of LA exposure (3 days post exposure). However, this study reported no alterations in adaptive immune responses or inflammatory cytokine production, either locally at the site of fiber deposition or systemically in serum [37]. We suspected that this lack of adaptive immune response was due to the short experimental duration. Therefore, in the present study, we investigated the effects of LGM2605 treatment using a 14-day model of LA exposure, which we predicted would allow sufficient time for adaptive immune activation.

Within the present study, LA exposure enhanced total immune cell influx to the site of fiber deposition and increased pro-inflammatory cytokine levels, both locally and systemically, at the 14-day time point. LGM2605 treatment reduced total immune cell influx in LA-exposed mice as well as decreased pro-inflammatory cytokine levels. LGM2605 also reversed the effects of LA on lymphocyte recruitment and B cell activation, measured as immunoglobulin class switching. Together, these data suggest a protective role of LGM2605 on LA-induced innate and adaptive immune responses. Further, we found no differences between male and female mouse responses to either LA exposure or LGM2605 treatment, Lastly, LGM2605 demonstrated protective effects against LA-induced guanine oxidation, which may be relevant for future long-term investigations as oxidative stress has been associated with many systemic diseases including cancers, chronic inflammation, and autoimmunity.

In this current study, we utilized i.p. fiber exposure and oral drug treatments. Although asbestos fiber exposures typically occur via inhalation, systemic fiber deposition has been noted [45]. The peritoneal cavity specifically is utilized as a surrogate for the pleural cavity for several reasons: (1) collection of pleural cavity tissue can be difficult, yielding few cells for subsequent analysis [10,39], (2) cellular composition of pleural and peritoneal cavities are similar, resulting in comparable inflammatory and cytotoxic reactions upon amphibole asbestos exposure [38], and (3) mice do not inhale asbestos fibers in the same way that humans do, thus necessitating fiber exposure via injection. Intratracheal fiber exposures require invasive surgery with potential side-effects, therefore making i.p. injections the preferred exposure pathway for short-term studies. Use of the Medigel cup for LGM2605 administration was also selected to limit invasive procedures like oral gavage, which tends to be stressful for mice, resulting in increased morbidity that may skew the observed physiological outcomes [46]. Oral gavage is also relatively time-consuming when utilized for daily treatments, making it undesirable for long-term dietary intervention studies. In contrast, drug consumption in Medigel cups is less stressful for mice, as it reduces total handling time; it also requires less time for daily treatments. To ensure dosing consistency, however, it is important that both mice and cups are weighed daily, altering the dosage amount per cage as needed [47]. Appropriate drug dosages are not guaranteed by this strategy, as gel consumption may vary between individual mice. However, the data presented here suggest that individual mouse consumption of LGM2605-containing gel was sufficient for hydration and weight gain, and that drug consumption provided significant protective effects against LA fiber exposure. Others have also noted oral administration of SDG results in systemic distribution of lignans [48] and reduced inflammation [49].

Overall, our results show a protective effect of LGM2605 against LA-induced innate immune responses. Specifically, LGM2650 attenuated LA-induced splenomegaly and innate immune cell recruitment and activation within the peritoneal cavity. Splenomegaly, or enlargement of the spleen, was previously shown to occur following LA exposure and attenuated by LGM2605 [37]. Increased spleen weights may serve as an indicator of immune activation since immune cells pass through the spleen when trafficking to the site of inflammation/infection. Splenomegaly is a well-documented feature of autoimmune disease, including rheumatoid arthritis (RA) and systemic lupus erythematosus (SLE) [50], which are commonly associated with LA exposure [51]. Treatment with LGM2605 + LA significantly reduced spleen weights that were enlarged by LA exposure, suggesting a reduction in immune activation. These findings were substantiated by flow cytometry data, which in general showed an increase in immune cell trafficking among LA-exposed mice that was countered by LGM2605 treatment.

In particular, we noted increased PMN and leukocyte numbers in the peritoneal cavity following LA exposure. PMN and macrophage recruitment occurs as part of the innate immune response; these cells are generally regarded as first responders in inflammation/infection and are important mediators of oxidative stress [52]. Treatment with LGM2605 + LA decreased the percentages of peritoneal PMN and macrophages back to control levels, demonstrating reduced trafficking of these cells to the site of fiber deposition. No differences between male and female mice were shown. Together, these data suggest that LGM2605 reduced immune cell recruitment induced by LA exposure. These finding corroborate our previous data suggesting LGM2605 limits very early immune activation and shows that this immune attenuation is sustained over a two-week time period.

To further investigate the effects of LA exposure and LGM2605 treatment on innate immune responses, we measured B1a cell populations. B1a cells are innate immune cells that may play a crucial role in LA-associated autoimmunity [39]. Exposure to LA fibers led to a significant increase in the percentages of both peritoneal (Figure 4) and splenic (Figure 5A) B1a cells, while treatment with LGM2605 + LA significantly reduced these populations back to control levels. B1a cells are long-lived, self-renewing lymphocytes that are found primarily in the peritoneal and pleural cavities [53]. They are largely responsible for the spontaneous secretion of circulating IgM referred to as “natural antibodies,” allowing these cells to respond rapidly to inflammation/infection without the need for prior immunization [54]. B1a cells can recognize self-antigens, which may serve in the clearance of apoptosis products. However, due to their autoreactivity, B1a cells have been linked to autoimmune disease in both human and animal models, playing a role in the development of such conditions as RA [55] and SLE [56]. B1a cells have been shown to respond to amphibole asbestos exposure by trafficking from the site of inflammation to the spleen and lymph nodes, where they may ultimately promote immune dysfunction [39]. Therefore, the observed reduction of B1a cells following oral administration of LGM2605 suggests that early LGM2605 treatment may protect against later development of LA-associated autoimmunity. Interestingly, female mice had a significantly higher splenic B1a cell population compared to males within the control and LA treatment groups (Figure 5B), suggesting physiologic differences in immune cell composition between male and female mice, as demonstrated previously [57]. Further research is necessary to elucidate the effect of LGM2605 alone on these B1a cell populations.

Previous in vivo studies have shown a decrease in both pleural and peritoneal B1a cells at 3 days post-LA exposure, indicative of B1a cell trafficking to secondary lymphatic organs [37,39]. However, recovery of pleural B1a cells was reported at 6 days post LA-exposure and trafficking of these cells to the spleen and lymph nodes peaked at about 7 days post-LA exposure [39]. In the present study, our results were consistent with these findings. The observed increase in peritoneal as well as splenic B1a cells suggests that B1a cells activated in the spleen may return to the site of local inflammation by 14 days post-LA exposure, allowing for a recovery of peritoneal B1a cell numbers. Follow-up studies are needed to more fully elucidate the effects of LA exposure on B1a cell populations over an extended treatment period and to understand the difference in B1a cell populations between males and females. Future long-term investigations should also evaluate alpha-4 integrin, a surface marker that controls B cell retention in the peritoneum, in order to confirm active trafficking of B1a cells [37]. Previously, alpha-4 integrin expression was shown to decrease on B1a cells following LA exposure, signifying a detachment of these cells from the peritoneal cavity and increased cell motility [39]. Although we did not measure alpha-4 integrin expression within the present study, it is possible that alpha-4 integrin may be downregulated at 14 days post-LA exposure, contributing to an accumulation of these cells within the peritoneal cavity.

LA exposure also attenuated adaptive immune responses, resulting in significant decrease in the percentage of total peritoneal B and T cells (Figure 6). However, total splenic B and T cells were unaltered by LA, suggesting that lymphocytes were not actively trafficking via the spleen. Instead, the depressed peritoneal B cell numbers may have instead resulted from macrophage activity. This finding is supported by a previous in vitro study showing that asbestos exposure stimulates macrophages to release TNF-α and IL-6, and that these cytokines in turn suppress B-cell proliferation [58]. Since both peritoneal macrophages and IL-6 production were significantly increased by LA in the present study (Figure 3 and Figure 7), this effect may further explain the observed decrease in total peritoneal B cells following LA exposure.

Total peritoneal T cell levels were also depressed following LA exposure, though no effects of treatment on Th cell levels were noted. While other studies demonstrate direct effects of various asbestos fibers on T cell activity (i.e., apoptosis, receptor expression [59,60]), similar effects have not been reported for LA fiber exposures. Previous studies show Th-cell suppression 3 days post-LA exposure [37]; long-term studies of LA exposures have shown no alterations in splenic or peritoneal T cell numbers at 7- or 8-months post-exposure [10]. These studies, coupled with our current data, suggest that Th cells experienced a recovery in their numbers by 14 days post-LA exposure, allowing for a return to control levels. Despite this apparent recovery, it is possible that early Th cell suppression (3 days post-exposure [37]) contributed to the observed suppression of peritoneal B cells since B cells rely on activation by Th cells. Although not evaluated in the present study, it is also possible that B and T cell maturation may vary in response to LA, despite limited changes in total cell numbers. Future studies are needed to evaluate levels of immature and mature lymphocytes following LA exposure and to provide additional insight into the effects of LA exposure on immune activation. Mechanistic studies should also be performed to assess the contribution of lymphocyte activity to LA-associated pathologies and to evaluate the role of LGM2605 in controlling this activity.

To further evaluate adaptive immune responses, immunoglobin isotypes were analyzed. LA exposure contributed to a significant decrease in the relative concentrations of IgG_1_ and IgG_2b_, while treatment with LGM2605 + LA restored these concentrations back to control levels (Figure 7). Murine IgG_1_ is functionally similar to human IgG_4_ [61], and we have previously noted low levels of IgG_4_ in serum of LA-exposed populations relative to other fiber exposures [55]. Both murine IgG_1_ and human IgG_4_ may possess immunosuppressive effects by inhibiting complement activation, thus protecting against inflammation and cell death [55]. Decreased levels of these antibodies may therefore suggest an increase in asbestos-induced cytotoxicity. LA exposure also contributed to a significant increase in the relative concentration of IgA (Figure 7). IgA is the most abundantly produced isotype in higher mammals and is commonly associated with mucosal surfaces, where it forms a first line of defense against inhaled or ingested pathogens. Notably, IL-6 has been shown to enhance IgA synthesis by promoting terminal differentiation of IgA-committed B cells [62], and we demonstrated that IL-6 concentrations were significantly increased by LA exposure (Figure 8). Furthermore, murine B1 cells in the peritoneal cavity are distinguished from other B cells by their preferential class switching to IgA under minimal stimulatory conditions, via an IL-6-independent pathway [63,64,65]. Murine cell line models have shown that LA induces increased surface expression of IgA among peritoneal B1a cells [58], indicative of isotype class switching. In the present study, we observed an increase in peritoneal B1a cells following LA exposure (Figure 4), suggesting a likely role of these cells in IgA production. Interestingly, a previous in vivo study reported an increase in peritoneal IgM at 3 days post-LA exposure, followed by a decrease in IgM to control levels by days 6–7 [39]. Because we observed an increase in IgA but no changes in IgM, this result indicates isotype class switching from IgM to IgA at 14 days post-LA exposure. Treatment with LGM2605 + LA reduced the relative concentration of IgA back to control levels, potentially by inhibiting LA-induced isotype class switching and effectively regulating immune responses.

Among all immunoglobulin isotypes examined in PLF, LA exposure consistently increased the ratio of κ/λ light chains (Table 1), corresponding to increased λ light chain production. The normal κ/λ ratio in human sera is approximately 1.5, while the normal κ/λ ratio for mice is approximately 19 [66], although a slightly different composition is expected in PLF. Any significant deviation from normal range may constitute an immune disturbance. Notably, disturbed κ/λ ratios have been described in various autoimmune conditions, including RA [67], and may serve as important prognostic factors. In addition, B1 cells in the peritoneal cavity are noted for their increased λ light chain production [68]. These cells are responsible for the secretion of IgM autoantibodies, and their numbers have been shown to increase within autoimmune animals [69]. The observed increase in λ light chain production at 14 days post-LA exposure—coupled with an increase in peritoneal B1a cells (Figure 4)—may therefore suggest increased autoimmune activity. Meanwhile, treatment with LGM2605 + LA returned κ/λ ratios to control levels, suggesting protective effects in the context of LA-associated autoimmunity. However, more research is necessary to determine the physiological relevance of κ/λ ratios in LA exposure models and their implications for later disease development.

To further investigate the effects of both LA exposure and LGM2605 treatment on inflammation, we measured cytokine levels in PLF and serum. LA exposure significantly increased the concentrations of the pro-inflammatory cytokines IL-6 and MCP-1 in PLF (Figure 8A), indicative of localized inflammation within the peritoneal cavity. Notably, IL-6 promotes differentiation of monocytes to macrophages [70], and MCP-1 is one of the major chemokines that regulates migration and infiltration of monocytes/macrophages as part of the inflammatory response [71]. These findings coincide with cellular flow cytometry data, which demonstrated an increase in peritoneal white blood cell influx following LA exposure (Figure 3). Treatment with LGM2605 + LA significantly reduced PLF concentrations of IL-6 and MCP-1 back to control levels, reducing inflammation and attenuating LA-induced inflammatory responses within the peritoneal cavity. These results are supported by the fact that LGM2605 treatment contributed to a reduction in peritoneal macrophages within LA-exposed mice. In addition, LA exposure significantly increased the concentration of IL-6 in serum (Figure 8B), indicative of systemic inflammation. Elevated serum levels of IL-6 have been implicated in autoimmune disease (i.e., RA, SLE) as well as other chronic, inflammatory conditions [71]. Additionally, these increases are similar to those reported in serum of asbestos-exposed human cohorts, suggesting clinical relevance of these changes [29]. Treatment with LGM2605 + LA significantly reduced the serum concentration of IL-6 back to control levels, suggesting beneficial effects in the context of LA-induced inflammation and associated disease pathologies. Treatment with LGM2605 + LA also increased the serum concentration of regulatory cytokine IL-10 compared to LA only (Figure 8B). Secreted by most cells of the immune system, IL-10 is regarded as both immunosuppressive and anti-inflammatory, as it suppresses the production of various pro-inflammatory cytokines and inhibits the antigen-presenting function of both macrophages and dendritic cells [72,73]. IL-10 may therefore combat chronic inflammation by mediating resolution of immune responses. Taken together, these results suggest that LA promotes both local and systemic inflammation by increasing pro-inflammatory cytokine production; LGM2605 not only counters these effects but also demonstrates likely immunosuppressive properties.

Lastly, we investigated the effects of LGM2605 on LA-induced oxidative stress. Asbestos exposure leads to generation of reactive oxygen species (ROS), which can oxidize macromolecules such as DNA, proteins, and lipids and lead to oncogenic transformations and chronic inflammation. Previous studies have shown that SDG reduces asbestos-associated oxidative stress in vivo and that LGM2605 blocks ROS and RNS in vitro [31,74]. However, effects of LGM2605 on LA-induced oxidative stress have not been examined. In the current study, we showed for the first time oxidative damage to DNA/RNA by LA exposure, as evidenced by the presence of oxidized guanine species in the peritoneal lavage fluid. Importantly, this was reduced by the action of LGM2605 to baseline levels (Figure 9). Oxidized guanine species are widely accepted biomarkers of exposure to diverse carcinogens including asbestos [75]. These data suggest that LGM2605 may be an effective preventative treatment for environmental exposures associated with oxidative stress responses.

In summary, LA exposure via i.p. injection promoted inflammatory responses and oxidative tissue damage in both male and female mice over a 14-day period. Oral treatment with LGM2605 ameliorated these responses, as measured by changes in immune cell trafficking, cytokine production, immunoglobulin isotype switching, and guanine species oxidation.

## 4. Materials and Methods

### 4.1. Animals

All experiments were approved by the Idaho State University Institutional Animal Care and Use Committee (IACUC). Ten-week-old female and male C57BL/6 mice (Jackson Laboratories, Bar Harbor, ME, USA) were housed three per cage in the Idaho State University Animal Care Facility with a 12 h light-dark cycle, constant temperature (22 °C) and humidity (45%), and ad libitum access to standard rodent chow.

### 4.2. LGM2605 Treatment

LGM2605 was prepared as previously described [76] and provided as a lyophilized aliquot by the University of Pennsylvania. Briefly, LGM2605 was synthesized from vanillin via secoisolariciresinol and glucosyl donor (perbenzoyl-protected trichloacetimidate under the influence of TMSOTf) through a concise route involving chromatographic separation of diastereomeric diglucoside derivatives (Chemveda Life Sciences, Inc., Hyderabad, India). Lyophilized samples of LGM2605 (100 mg/vial) were reconstituted with sterile PBS to produce stock solution of 33.3 mg/mL. Mice were treated daily with approximately 100 mg/kg LGM2605 (see Section 2.1) using Medigel Sucralose Clear H_2_O cups (Thermo Fisher Scientific, Waltham, MA, USA) as a delivery mechanism. Cups were prepared according to manufacturer recommendations. Briefly, reconstituted LGM2605 was added to room temperature cups by puncturing the foil lid. Lids were then taped shut and vigorously shaken for 30 s to disperse liquid LGM2605. A drop of food dye was added to the cup prior to mixing to visualize even distribution through the gel. Cups were changed daily so freshly prepared LGM2605 was administered. Medigel consumption was monitored daily by weighing cups at the start and end of each 24-h period. Amount of LGM2605 added to each cup was calculated daily based on average Medigel consumption and weight of the mice in each cage for each day (see Section 2.1). Control mice were given Medigel cups supplemented with equal volume of sterile PBS. Figure 1 illustrates assignment of mice to treatments (A) as well as treatment schedule (B).

### 4.3. Mineral Fibers (LA)

Libby amphibole (LA) asbestos was provided by Dr. Jean Pfau from Montana State University, provided to her by the United States Environmental Protection Agency (EPA) as a composite sample of asbestos-rich rock samples collected from multiple sites in the W.R. Grace mine outside of Libby (MT, USA). The LA sample was previously characterized as described [1,77,78]. LA fibers were not elutriated.

### 4.4. LA Exposure

LA fibers were prepared as previously described [77]. Briefly, fibers were prepared as 1 mg/mL suspensions in sterile phosphate buffered saline (PBS, pH 7.4) and sonicated (Branson Ultrasonics, Danbury, CT, USA) for fifteen minutes prior to use to minimize aggregation. Fiber solutions were boiled for 1 h to inactivate any bacterial contaminants [79] and then tested for endotoxin. Endotoxin testing of fiber suspensions was performed prior to instillations using the ToxinSensor™ Chromogenic LAL Endotoxin Assay Kit (GenScript Biotech Corp., Piscataway, NJ, USA), following the manufacturer′s protocol. Samples included phosphate-buffered saline (PBS) and suspended LA (1.0 mg/mL) in sterile PBS tested against a standard curve provided with the kit. Briefly, all samples were added to endotoxin-free tubes along with 100 µL of LAL and incubated at 37 °C for 6 min. Stop solution and color stabilizers were added to each tube and then 200 µL of each solution moved to a 96 well plate. Absorbance was read in duplicate for each sample at 545 nm. Endotoxin was detected at 0.071 EU/mL, which is below the acceptable lower limit for animal exposures. Mice were subsequently exposed to LA fibers via intraperitoneal injection using a 25-gauge needle with 200 µL, thus giving 200 µg per mouse in a single bolus dose.

### 4.5. Tissues Harvest

At the experimental endpoint, animals were euthanized using CO_2_ asphyxiation followed by cardiac puncture for blood collection. Blood was allowed to clot and then centrifuged to collect serum. Serum was stored at −20 °C until use. Peritoneal lavage fluid (PLF) was collected by first injecting four mL of sterile PBS with 5% fetal bovine serum (FBS) into the intact peritoneal cavity, gently agitating the peritoneal cavity, and then aspirating three mL using a 21-gauge needle. PLF was centrifuged, and the cells were prepared for flow cytometry. The resulting cell-free PLF was aliquoted and frozen for further analyses. Spleens were also harvested, weighed, and then minced and prepared as single cell suspensions as previously described [6], including a brief wash in Red Blood Cell Lysis solution (eBioscience/ThermoFisher Scientific, Waltham, MA, USA) to select for white blood cells. The splenocytes were counted using a Cellometer Auto T4 (Nexcelom Bioscience, Lawrence, MA, USA), and 1 million cells from each sample were placed in separate tubes containing 100 µL PBS with 3% Bovine Serum Albumin as blocking agent. Sets of cells were stained with BD Biosciences (San Jose, CA, USA) antibodies as follows:

- B cells: CD19 (PE) or IgM (PerCP Cy5.5)
- B1a B cells: IgM^pos^ (PerCP Cy5.5), CD5^pos^ (APC), CD23^neg^ (PE)
- T cells: CD3 (FITC) ^pos^
- Helper T cells: CD3 (FITC)^pos^, CD4 (PE)^pos^
- Polymorphonuclear cells (PMN): Ly-6G (APC)

Cells were stained for 30 min at 4 °C and then washed with 1 mL ice cold PBS, twice. Staining was analyzed on a FACS Calibur flow cytometer using Cell Quest software (BD Biosciences). Isotype control antibodies (BD Biosciences) determined background staining, and less than 1% of these controls was allowed in the M1 gate for percent positive. Peritoneal white blood cells were separated into three distinct populations based on forward and side scatter, previously identified as lymphocytes (small, low side scatter), macrophages (larger, higher side scatter), and granulocytes (very high side scatter). Tight polygonal regions were used (instead of quadrants) to identify the subpopulations. By staining lymphocytes and PMNs for specific markers, cells in the macrophage gate were confirmed as being negative for CD19, CD3, and Ly-6G. The major subsets within the lymphocyte gate were identified based on the antibodies listed above.

### 4.6. Cytokine Detection

Cytokine levels in PLF and serum were measured using the Cytometric Bead Array (CBA) Mouse Inflammation Kit (BD Biosciences). The following cytokines were measured using CBA with flow cytometry: interleukin-6 (IL-6), interleukin-10 (IL-10), monocyte chemoattractant protein-1 (MCP-1), interferon-γ (IFN-γ), tumor necrosis factor (TNF), and interleukin-12p70 (IL-12p70). The CBA procedure was carried out according to the manufacturer’s instructions. Briefly, a standard curve was prepared for each cytokine assayed. PLF samples were concentrated three-fold using a CentriVap concentrator (Labconco, Kansas, MO, USA); serum samples were not concentrated. 50 μL of concentrated PLF, serum, or prepared standard were then mixed with 50 µL of capture beads, mixed with PE detection reagent supplied with the kit, and incubated in the dark for 2 h at room temperature. Samples were washed, supernatant discarded, and pellets resuspended and analyzed on a FACS Calibur flow cytometer using Cell Quest software (BD Biosciences). Instrument set-up was done using the instrument set-up beads provided with the kit.

### 4.7. Immunoglobulin Isotyping

Immunoglobulin isotypes in PLF were evaluated using the Cytometric Bead Array (CBA) Mouse Immunoglobulin Isotyping Kit (BD Biosciences). The following were measured: IgG_1_, IgG_2a_, IgG_2b_, IgG_3,_ IgA, IgM, and IgE. κ and λ light chains were also evaluated for each isotype. The CBA procedure was carried out according to the manufacturer’s instructions. Briefly, 50 μL of concentrated PLF or prepared standard mouse Ig standards (2.5 µg/mL) were mixed with 50 µL of capture beads, and centrifuged. Pellets were resuspended, mixed with PE detection reagent, and incubated in the dark for 15 min at room temperature. Samples were washed and analyzed on a FACS Calibur flow cytometer using Cell Quest software (BD Biosciences) following instrument set-up using the instrument set-up beads provided with the kit.

### 4.8. Detection of DNA/RNA Oxidative Damage in PLF

Levels of markers of DNA/RNA oxidative damage, 8-hydroxy-2′-deoxyguanosine (8-OHdG) from DNA, 8-hydroxyguanosine from RNA, and 8-hydroxyguanine from either DNA or RNA, were determined in the PLF using an DNA/RNA Oxidative Damage ELISA kit (Cayman Chemical, Ann Arbor, MI, USA) according to the manufacturer’s protocol. The effect of LGM2605 on levels of oxidized guanine species was determined in murine PLF samples at day 14 post-LA exposure. This high sensitivity ELISA kit recognizes not only 8-OHdG, but other oxidized guanine species as well, such as 8-hydroxyguanine and 8-hydroxyguanosine [80]. PLF samples from male mice were run undiluted and PLF samples from female mice were run diluted, all in duplicate. Data are reported as the mean fold change in the levels of oxidized guanine species from their respective, non-LA-exposed controls, in PLF.

### 4.9. Statistical Analysis

All data were tested for normality prior to statistical analysis. Data for all cell types and inflammatory markers were analyzed using two-way analysis of variance (ANOVA) to test for the main effects of treatment and sex, along with the interaction between these variables, on study outcome measures. If the overall F-test was statistically significant, Tukey’s HSD post hoc tests were conducted to determine significant differences between LA exposure groups (control versus LA) and among treatment groups (no LGM2605 versus LGM2605). Results are reported as mean ± the standard error of the mean (SEM). Statistically significant differences were determined with *p*-value < 0.05. Asterisks shown in figures indicate significant differences between LA exposure groups (control versus LA) (* *p* < 0.05, ** *p* < 0.01, *** *p* < 0.001 and **** *p* < 0.0001). # shown in figures indicate significant differences between LA-exposed mice treated with or without LGM2605 (LA versus LA + LGM2605) (# *p* < 0.05, ## *p* < 0.01, ### *p* < 0.001 and #### *p* < 0.0001). One-way ANOVA was utilized to specifically examine sex effects within individual treatment groups and/or treatment effects subdivided by gender. Data analyses were performed using R statistical analysis software (Version 4.0.2, R Foundation for Statistical Computing, Vienna, Austria) and GraphPad Prism version 6.00 for Windows (GraphPad Software, La Jolla, CA, USA, www.graphpad.com, accessed on 7 October 2021).

## Figures and Tables

**Table 1:** PLF immunoglobulin isotypes by cytometric bead array. Immunoglobulin concentrations are presented as relative concentration ± standard error, using saline control as standard. κ/λ ratios are presented as mean concentration κ antibody/mean concentration λ antibody for each isotype. In general, LA treatment decreased κ/λ ratios; treatment with LGM2605 + LA restored κ/λ ratios to control levels. Data were analyzed via two-way ANOVA to analyze overall treatment effects (sex differences not shown).

	Control	LGM2605 Only	LA Only	LGM2605 + LA	*p*-Value Treatment
IgG_1_ conc.	1.00 ± 0.10	1.19 ± 0.09	0.53 ± 0.08	1.13 ± 0.16	<0.001
κ/λ ratio	93.18	91.58	32.44	81.19	
IgG_2a_ conc.	1.00 ± 0.08	1.16 ± 0.10	0.72 ± 0.11	1.05 ± 0.10	0.023
κ/λ ratio	63.00	89.61	24.76	32.95	
IgG_2b_ conc.	1.00 ± 0.08	1.06 ± 0.07	0.53 ± 0.04	1.13 ± 0.13	<0.001
κ/λ ratio	89.32	114.34	16.33	93.87	
IgG_3_ conc.	1.00 ± 0.09	1.27 ± 0.06	0.85 ± 0.09	1.21 ± 0.15	0.036
κ/λ ratio	12.42	16.77	7.68	17.19	
IgA conc.	1.00 ± 0.14	0.86 ± 0.03	1.68 ± 0.26	0.94 ± 0.16	<0.01
κ/λ ratio	7.24	6.97	0.98	5.38	
IgM conc.	1.00 ± 0.06	1.29 ± 0.14	1.14 ± 0.07	1.16 ± 0.09	n.s.
κ/λ ratio	32.93	64.81	8.27	15.80	
IgE conc.	1.00 ± 0.10	1.83 ± 0.48	1.45 ± 0.18	1.20 ± 0.24	0.032
κ/λ ratio	2.37	6.92	0.37	2.67	

**Table 2:** PLF cytokines by cytometric bead array. Data are presented as mean concentration in pg/mL ± std error. Data were analyzed by two-way ANOVA (for overall treatment effects) and by one-way ANOVA (for treatment effects subdivided by sex).

	Control	LGM2605 Only	LA Only	LGM2605 + LA	*p*-Value Treatment
IL-6	0.36 ± 0.17	0.89 ± 0.42	16.84 ± 4.16	0.22 ± 0.14	<0.0001
Female	0.26 ± 0.12	0.41 ± 0.14	12.86 ± 1.77	0.11 ± 0.03	<0.001
Male	0.46 ± 0.22	1.62 ± 0.59	18.03 ± 5.89	0.33 ± 0.20	<0.001
MCP-1	0.77 ± 0.2	1.04 ± 0.20	1.88 ± 0.29	0.65 ± 0.10	<0.001
Female	0.65 ± 0.18	0.91 ± 0.20	1.90 ± 0.32	0.55 ± 0.09	0.026
Male	0.90 ± 0.22	1.31 ± 0.22	1.63 ± 0.29	0.74 ± 0.12	n.s.

**Table 3:** Serum cytokines by cytometric bead array. Data are presented as mean concentration in pg/mL ± std error. Data were analyzed by two-way ANOVA (for overall treatment effects) and by one-way ANOVA (for treatment effects subdivided by sex).

	Control	LGM2605 Only	LA Only	LGM2605 + LA	*p*-Value Treatment
IL-6	5.47 ± 0.82	4.25 ± 0.38	7.87 ± 1.06	4.38 ± 0.50	<0.01
Female	5.28 ± 1.42	3.67 ± 0.37	7.04 ± 1.30	5.26 ± 0.81	n.s.
Male	5.66 ± 0.97	4.83 ± 0.65	8.71 ± 1.73	3.49 ± 0.31	0.036
IL-10	15.83 ± 1.35	18.46 ± 1.65	13.09 ± 1.33	21.31 ± 2.59	0.016
Female	17.69 ± 2.02	17.03 ± 1.94	11.86 ± 0.19	25.29 ± 3.85	0.014
Male	13.97 ± 1.59	19.89 ± 2.71	14.31 ± 1.57	16.54 ± 2.00	n.s.
MCP-1	9.30 ± 1.37	6.50 ± 0.46	5.41 ± 0.42	6.47 ± 0.47	<0.01
Female	9.19 ± 2.16	7.35 ± 0.54	5.18 ± 0.59	6.60 ± 0.54	0.045
Male	9.41 ± 1.89	6.47 ± 0.47	5.64 ± 0.63	6.31 ± 0.88	n.s.

## Data Availability

Not applicable.
